# Astaxanthin Attenuates Early Acute Kidney Injury Following Severe Burns in Rats by Ameliorating Oxidative Stress and Mitochondrial-Related Apoptosis

**DOI:** 10.3390/md13042105

**Published:** 2015-04-13

**Authors:** Song-Xue Guo, Han-Lei Zhou, Chun-Lan Huang, Chuan-Gang You, Quan Fang, Pan Wu, Xin-Gang Wang, Chun-Mao Han

**Affiliations:** 1 Department of Burns, Second Affiliated Hospital, School of Medicine, Zhejiang University, 88 Jiefang Road, Hangzhou 310009, Zhejiang, China; E-Mails: 11318225@zju.edu.cn (S.-X.G.); zhlei288@zju.edu.cn (H.-L.Z.); 2314053@zju.edu.cn (C.-G.Y.); wuzhiqiujin@zju.edu.cn (P.W.); wangxingang8157@zju.edu.cn (X.-G.W.); 2 Department of Plastic Surgery, First Affiliated Hospital, School of Medicine, Zhejiang University, 79 Qingchun Road, Hangzhou 310000, Zhejiang, China; E-Mail: 21418262@zju.edu.cn; 3 Department of Plastic Surgery, Binjiang Branch, Second Affiliated Hospital, School of Medicine, Zhejiang University, 1511 Jianghong Road, Hangzhou 310000, Zhejiang, China; E-Mail: 21218125@zju.edu.cn

**Keywords:** astaxanthin, acute kidney injury, burn, oxidative stress, apoptosis, mitochondria

## Abstract

Early acute kidney injury (AKI) is a devastating complication in critical burn patients, and it is associated with severe morbidity and mortality. The mechanism of AKI is multifactorial. Astaxanthin (ATX) is a natural compound that is widely distributed in marine organisms; it is a strong antioxidant and exhibits other biological effects that have been well studied in various traumatic injuries and diseases. Hence, we attempted to explore the potential protection of ATX against early post burn AKI and its possible mechanisms of action. The classic severe burn rat model was utilized for the histological and biochemical assessments of the therapeutic value and mechanisms of action of ATX. Upon ATX treatment, renal tubular injury and the levels of serum creatinine and neutrophil gelatinase-associated lipocalin were improved. Furthermore, relief of oxidative stress and tubular apoptosis in rat kidneys post burn was also observed. Additionally, ATX administration increased Akt and Bad phosphorylation and further down-regulated the expression of other downstream pro-apoptotic proteins (cytochrome c and caspase-3/9); these effects were reversed by the PI3K inhibitor LY294002. Moreover, the protective effect of ATX presents a dose-dependent enhancement. The data above suggested that ATX protects against early AKI following severe burns in rats, which was attributed to its ability to ameliorate oxidative stress and inhibit apoptosis by modulating the mitochondrial-apoptotic pathway, regarded as the Akt/Bad/Caspases signalling cascade.

## 1. Introduction

Acute kidney injury (AKI) is a major common and devastating complication in severely burned patients (total body surface area (TBSA) ≥20%). The incidence of acute renal dysfunction in critical care burn patients ranges from 15% to 40% [1], and early AKI (appearing during the first five days post burn) is associated with poor prognosis and a high mortality (approximately 80%) [2,3,4,5]. The pathogenesis of early AKI may be attributed to intravascular hypovolaemia, systemic vasoconstriction, early organ dysfunction or myoglobinuria, resulting from oxidative stress or apoptosis [6,7,8]. The pathophysiological effects of mitochondrial and cellular reactive oxygen species (ROS) have been suggested to be involved in the development of local or distant organ injuries in some diseases and traumatic conditions, such as severe burns, haemorrhagic brain injury, diabetes, and sepsis [9,10,11,12,13,14,15]. ROS-mediated oxidative stress is involved in the development of renal dysfunction followed by AKI or other diseases, which may result in disruptions in cellular functions and induced mitochondrial-related apoptosis via various free radicals [16,17]. Therefore, based on increased research evidence, early and timely interventions for oxidative stress and secondary apoptosis in renal tissue may present a potential protective avenue for severe burn-induced AKI [18].

In the mitochondrial-apoptotic pathway, phosphoinositide 3-kinase (PI3K)/ protein kinase B (Akt) plays a critical role in anti-apoptotic/survival signalling to affect the activation of downstream apoptosis-related proteins, such as Bad (Bcl-xL/Bcl-2-associated death promoter homologue), cytochrome c and caspase 9/3 [19,20,21,22]. It has been previously verified that phosphorylated Akt (p-Akt) is down-regulated with progressive deterioration of renal function in the early stages after severe burns, combined with an increase in tubular cell apoptosis [18]. Additionally, the PI3K/Akt pathway is also involved in the injury mechanisms of several traumatic insults based on its effect on tissue cell apoptosis [23,24].

Astaxanthin (3,3'-dihydroxy-b, b'-carotene-4,4'-dione, ATX) is a natural carotenoid with more robust anti-oxidative effects than other carotenoids based on quenching of free radicals, which are widely distributed in marine organisms, such as algae, crustaceans, salmon, shrimp, and crab [25,26]. In previous studies, ATX exhibited protective effects against oxidative stress-induced cell or tissue damage based on the results of *in vitro* and *in vivo* experiments [27,28]. It has also been reported that ATX protects against oxidative stress, inflammation, and apoptosis of high-glucose-exposed proximal tubular epithelial cells and diabetic nephropathy rats [29,30]. Recently, Qiu X *et al.* demonstrated that pretreatment of ATX could protect against oxidative stress induced toxicity in tubular epithelial cells and attenuate ischemia/reperfusion (I/R) induced renal injury in mice via reducing oxidative stress, inflammation and tubular apoptosis [31]. Furthermore, the modulation of mitochondrial pathways, such as the PI3K/Akt/Bad signalling pathway, contributed to the therapeutic effect of ATX on an animal model of subarachnoid haemorrhage, colon carcinogenesis, obesity, cerebral or myocardial ischemia/reperfusion injury [23,32,33,34,35]. In addition, there is no adverse effect reported in prior clinical trials [12,36]. Given the important roles of oxidative stress and mitochondria-related apoptosis in severe burn-induced early AKI, we hypothesized the possible protection of ATX on early severe burn-induced AKI and explored the dose-dependent effect and potential mechanisms of ATX action in regulating oxidative stress and mitochondrial-related apoptosis.

## 2. Results

### 2.1. ATX Attenuates the Histological and Functional Deterioration of Severely Burned rat Kidneys

**Figure 1:**
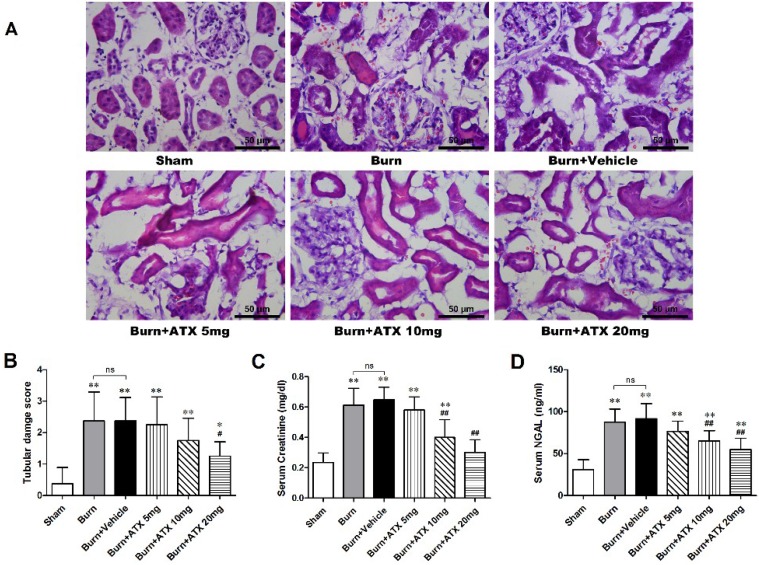
Histological and serum evaluations of renal function in the early stages after severe burn and ATX application. Representative HE-stained images of rat renal tissues are shown for all groups (*n* = 12 per group) at a magnification of 200× (**A**). Furthermore, the tubular damage scores provided quantitative verification (*n* = 8 per group) (**B**). Random-tested serum Cr (**C**) and NGAL (**D**) levels showed similar substantial elevations in the burn and vehicle groups at 24 h post burn, which indicated burn-induced early renal dysfunction, and ATX showed a dose-dependent effect on decreasing burn-induced elevations in serum Cr and NGAL levels (*n* = 8 per group). The results are expressed as the mean ± SD. * *p* < 0.05, ** *p* < 0.01, *vs.* sham; ^#^ *p* < 0.05, ^##^ *p* < 0.01, *vs.* burn + vehicle; ^ns^ *p* > 0.05.

Hematoxylin and eosin (HE) stained slices were analysed via the histological examination (Figure 1A). The tubular damage scores significantly increased in the burn and burn + vehicle groups at 24 h post burn, whereas the levels of serum creatinine (Cr) and neutrophil gelatinase-associated lipocalin (NGAL) were similarly elevated in the two groups (Figure 1B–D). With ATX administration, the 20 mg/kg group presented a significant decrease in the tubular damage score, compared with burn groups, although the tubular damage score in the 5 mg/kg and 10 m/kg groups declined slightly (Figure 1B). Regarding the serum Cr and NGAL levels, we found a dose-dependent decline in ATX administration groups, and the 20 mg/kg ATX groups exhibited the most remarkable change (Figure 1C,D).

### 2.2. ATX Ameliorates Severe Burn-Induced Oxidative Stress in Rat Renal Tissue

**Figure 2:**
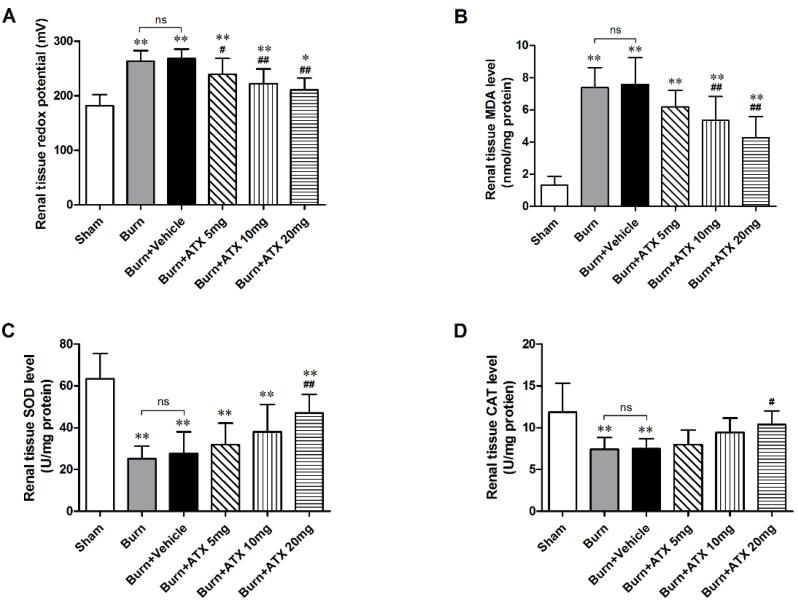
Assessment of oxidative stress and inner antioxidant enzyme levels in rat kidneys post burn and ATX application. After the burn insults, the levels of ORP and MDA in the burn and vehicle groups increased significantly, whereas the activities of the inner antioxidant enzymes (SOD and CAT) decreased remarkably. Following various doses of ATX, a marked reduction in the ORP and MDA levels and elevated activities of inner antioxidant enzymes were observed in the 20 mg/kg groups. The sample size was *n* = 8 for each group. The results are expressed as the mean ± SD. * *p* < 0.05, ** *p* < 0.01, *vs.* sham; ^#^ *p* < 0.05, ^##^ *p* < 0.01, *vs.* burn + vehicle; ^ns^ *p* > 0.05.

As a direct indicator, the oxidation-reduction potential (ORP) value reflects the redox status *in vivo*. After burn injury, the burn and burn + vehicle groups displayed similarly significant elevations in the renal tissue ORP values compared with that of the sham group (Figure 2A). All of three ATX management groups exhibited marked reductions in the ORP value, and the lowest values occurred in the burn + ATX20 mg/kg group (Figure 2A). Oxidative stress originates from an imbalance between oxidative and antioxidant effects *in vivo*, which can cause severe oxidative damage via various free radicals, such as ROS and reactive nitrogen species (RNS). ROS routinely cause lipid peroxidation tissue injury. The increased malondialdehyde (MDA, as an indicator of lipid peroxidation) levels in rats induced by severe burns are considered a marker of lipid peroxidation and oxidative stress (Figure 2B). ATX administration significantly decreased the elevations in the MDA levels in the rat renal tissues at 10 and 20 mg/kg groups, whereas 5 mg/kg ATX did not cause a marked decline (Figure 2B). The extensive burn injury also reduced the activities of inner antioxidant enzymes (Figure 2C,D). Burn insults substantially decreased the activities of renal tissue superoxide dismutase (SOD) and catalase (CAT), whereas high-dose (20 mg/kg) ATX significantly increased the activities of two antioxidant enzymes at 24 h after burn (Figure 2C,D). Taken together, these data indicate that ATX significantly improves burn-induced oxidative damage in rat kidneys.

### 2.3. ATX Relieves Burn-Induced Tubular Apoptosis in Rats

**Figure 3:**
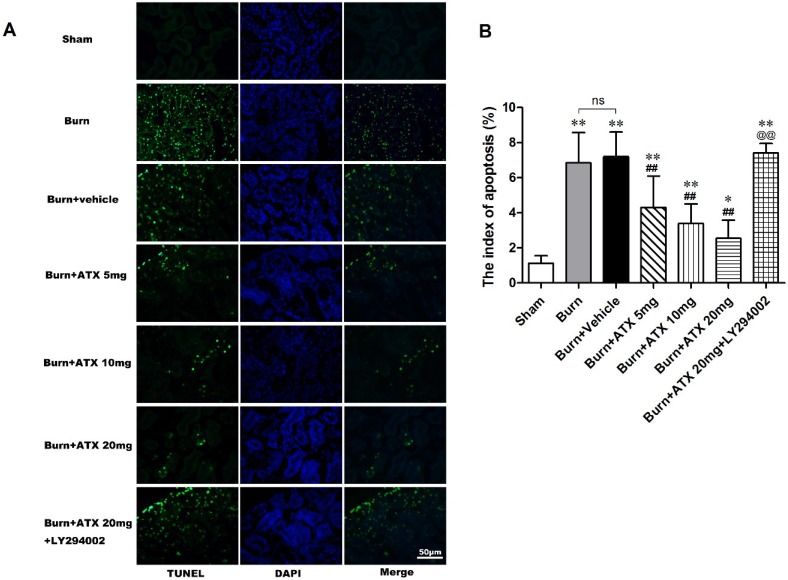
Effect of ATX on tubular apoptosis in rats post burn. Representative TUNEL stained images are shown for the various groups (**A**). The burn insults caused significant increases in green-fluorescent labelled nuclei in renal tubular cells of both burn and vehicle groups. The administration of ATX at different doses significantly decreased the number of green-fluorescent labelled nuclei post burn (**B**). LY294002 abolished the protective effect of ATX on apoptotic tubular cells (**B**). The indices of apoptosis in the different groups were consistent with the TUNEL staining results. The most effective ATX dose was 20 mg/kg. The sample size was *n* = 8 for each group. The results are expressed as the mean ± SD. * *p* < 0.05, ** *p* < 0.01, *vs.* sham; ^##^ *p* < 0.01, *vs.* burn + vehicle; ^@@^ *p* < 0.01, *vs.* burn + ATX 20 mg/kg group; ^ns^ *p* > 0.05.

Terminal deoxynucleotidyl transferase-mediated dUTP nick-end labelling (TUNEL) staining was applied to detect tubular apoptosis in the rat kidneys after severe burns. This index of apoptosis was introduced into the quantitative assessment. ATX at three doses ameliorated burn-induced renal tissue apoptosis (TUNEL-positive cells) after a burn, and the burn + ATX 20 mg/kg group showed the most robust effect (Figure 3A). In addition, the pretreatment of LY294002, an inhibitor of PI3K, reverses the effect of ATX on tubular apoptosis (Figure 3A). The indices of apoptosis significantly increased in the burn and burn + vehicle groups at 24 h after the burn insults, whereas ATX adminstration markedly reduced these increases (Figure 3B). LY294002 treatment significantly increased the index of apoptosis in rat kidney after a burn, even when 20 mg/kg ATX was administered (Figure 3B).

### 2.4. ATX Dose-Dependently Increases the Distribution and Protein Expression of p-Akt and p-Bad in Rats with Burns

**Figure 4:**
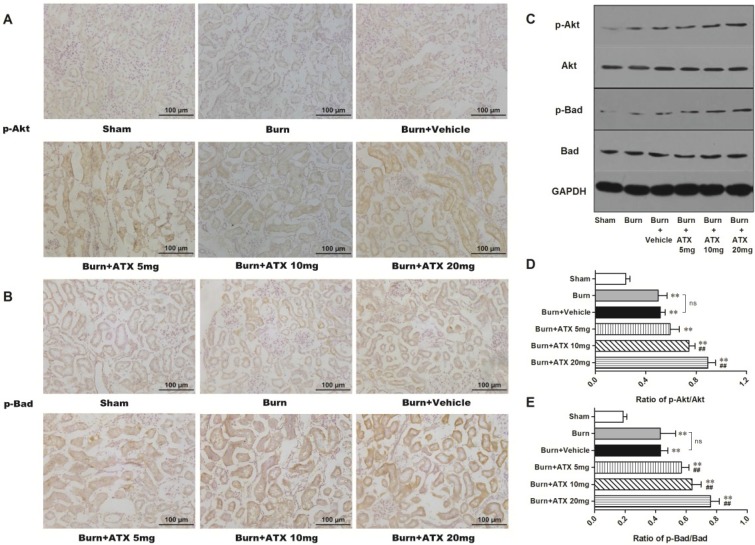
Effect of ATX on the distribution and protein expression of p-Akt and p-Bad in the kidneys of burned rats. As shown in (**A**,**B**), representative graphs show that severe burn insults resulted in reactive elevations in p-Akt or p-Bad positive staining in renal tubular cells as a protection against early AKI, compared with the sham group (*n* = 12 per group). With changes in the ATX doses, the number of positive-stained tubular cells increased further (*n* = 12 per group). The results of western blotting exposed similar tendencies of *p*-Akt or p-Bad protein expression post burn or ATX application (**C**–**E**) (*n* = 6 per group). The results are expressed as the mean ± SD. ** *p* < 0.01, *vs.* sham; ^##^ *p* < 0.01, *vs.* burn + vehicle; ^ns^ *p* > 0.05.

The expression and distribution of p-Akt and p-Bad were detected via IHC staining in rat kidneys after burn injury. Although more positive stained tubular cells appeared in the burn group and the burn + vehicle groups, compared with those of sham group, ATX further increased p-Akt and p-Bad immunoreactivity in the renal tubules of burned rats (Figure 4A,B). The most numerous p-Akt or p-Bad-positive cells were observed in the ATX 20 mg/kg groups (Figure 4A,B). The results of western blotting also identified the above changes in IHC staining (Figure 4C). Compared with the sham group, the renal tissue expression of p-Akt and p-Bad significantly increased after burn, whereas the vehicle group presented similar changes (Figure 4D,E). However, the expression of p-Akt and p-Bad was up-regulated gradually with elevated doses of ATX compared with the burn + vehicle group, and peaked at the 20 mg/kg dose (Figure 4D,E). These above results suggested that ATX administration further dose-dependently induces Akt and Bad phosphorylation in rat kidneys after burn insults.

### 2.5. Effects of ATX on Pro-Apoptotic Proteins of Mitochondrial Pathway

**Figure 5:**
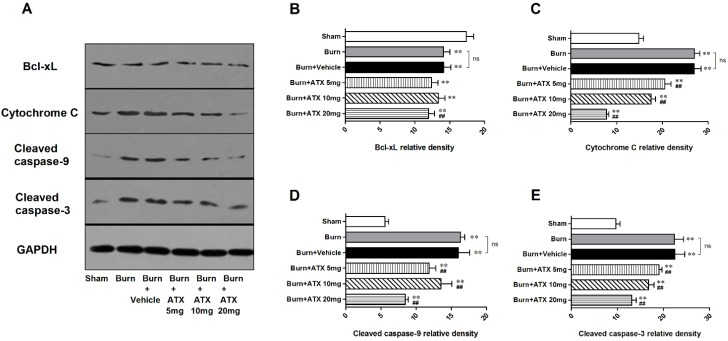
Expression changes in mitochondria pro-apoptotic proteins post burn with ATX administration. Although the expression of Bcl-xL decreased significantly post burn, all doses of ATX did not increase declined Bcl-xL levels (**A**,**B**). With regard to cytochrome c and cleaved caspase-3/9, burn insults caused obvious elevations in their protein expression in both burn and vehicle groups (**A**,**C**–**E**). ATX administration remarkably reduced the changes of protein expression, and the 20 mg/kg dose was the most effective (**A**,**C**–**E**). The sample size was *n* = 6 for each group. The results are expressed as the mean ± SD. ** *p* < 0.01, *vs.* sham; ^##^ *p* < 0.01, *vs.* burn + vehicle; ^ns^ *p* > 0.05.

As a binding protein with transferred Bad to construct a pro-apoptotic complex in the mitochondria, the renal expression of B-cell lymphoma (Bcl)-xL significantly decreased in the five groups at 24 h after burn. However, compared with the vehicle group, ATX administration slightly reduced the protein expression of Bcl-xL regardless of doses (Figure 5A,B). With regard to cytochrome c and activated caspase-3/9 (cleaved caspase-3/9), we observed similar up-regulation in renal samples of burn and burn + vehicle groups at 24 h post burn (Figure 5A,C–E). ATX application significantly ameliorated the increased expression of cytochrome c and cleaved caspase-3/9 (Figure 5A,C–E). The 20 mg/kg ATX dose exhibited the strongest effect on reducing the expression of these pro-apoptotic proteins (Figure 5A,C–E).

### 2.6. LY294002 Reverses the Effects of ATX on Renal Mitochondria Related Apoptosis after Burn

**Figure 6:**
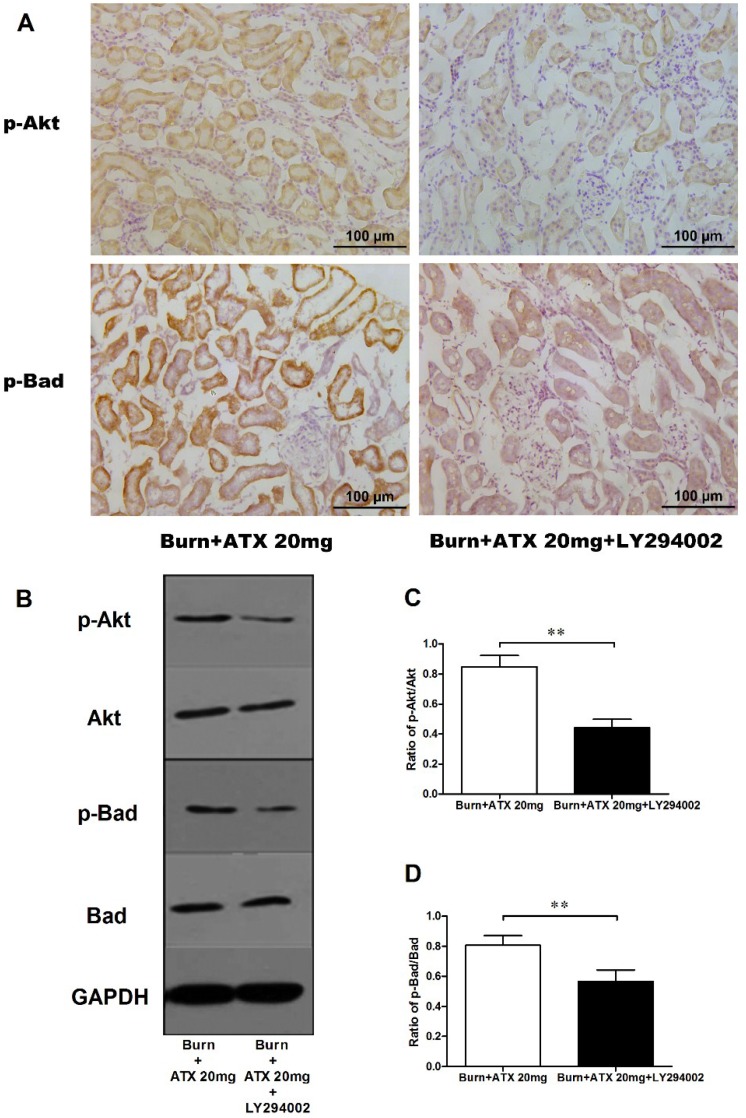
Effect of LY294002 on Akt and Bad phosphorylation in the kidneys of severe-burned rats with ATX management. Representative staining images of burn + 20 mg/kg ATX and burn + 20 mg/kg ATX + LY294002 groups are shown in (**A**) (*n* = 12 per group). With LY294002 pretreatment, the distribution of both p-Akt and p-Bad stained tubular cells decreased substantially. The results of western blotting indicated that LY294002 significantly reduced ATX-induced phosphorylation of p-Akt and p-Bad (*n* = 6 per group) (**B**–**D**). The results were expressed as the mean ± SD. ** *p* < 0.01.

**Figure 7:**
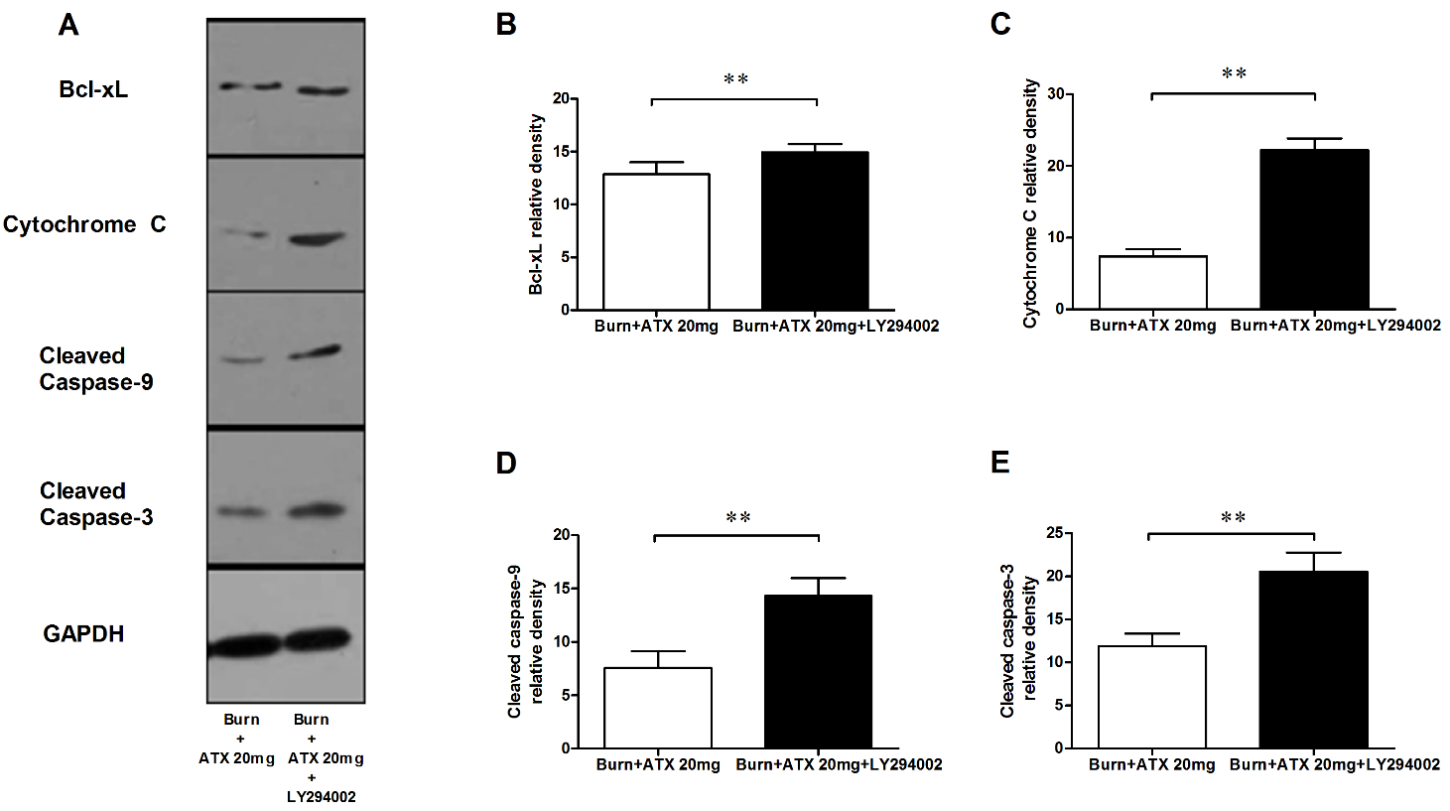
Effect of LY294002 on the renal expression of mitochondria pro-apoptotic proteins after ATX administration. The representative results of western blotting are shown in (**A**) (*n* = 6 per group). The analytic outcomes show that LY294002 pretreatment significantly increased the ATX-decreased protein expression of Bcl-xL (**B**); cytochrome c (**C**); and cleaved caspase 3/9 (**D**,**E**), compared with those of the burn + 20 mg/kg ATX group. The results are expressed as the mean ± SD. ** *p* < 0.01.

Pretreatment with specific the PI3K inhibitor, LY294002, was used to interrogate the possible mechanisms of ATX on early AKI after severe burn, which might be mediated by its regulation of the Akt/Bad/Caspase signalling cascade. LY294002 remarkably decreased the high-dose ATX (20 mg/kg) induced elevated distribution and expression ratio of p-Akt/Akt and p-Bad/Bad (representing the extent of Akt and Bad activation) in the kidneys of severely burned rats, based on the results of the IHC staining and western blotting (Figure 6A–D). Moreover, LY294002 also reversed the expression changes in mitochondrial-apoptosis related proteins (Bcl-xL, cytochrome c, caspase-3/9) mediated by ATX (20 mg/kg) treatment (Figure 7A–E). All of these indicated that the protective effect of ATX on early AKI after severe burn could be abolished by the inhibitory effect of LY294002 on the PI3K/Akt/Bad signalling pathway, and also indirectly verified the important role of the mitochondrial-dependent pathway in the therapeutic mechanisms of ATX.

## 3. Discussion

**Figure 8:**
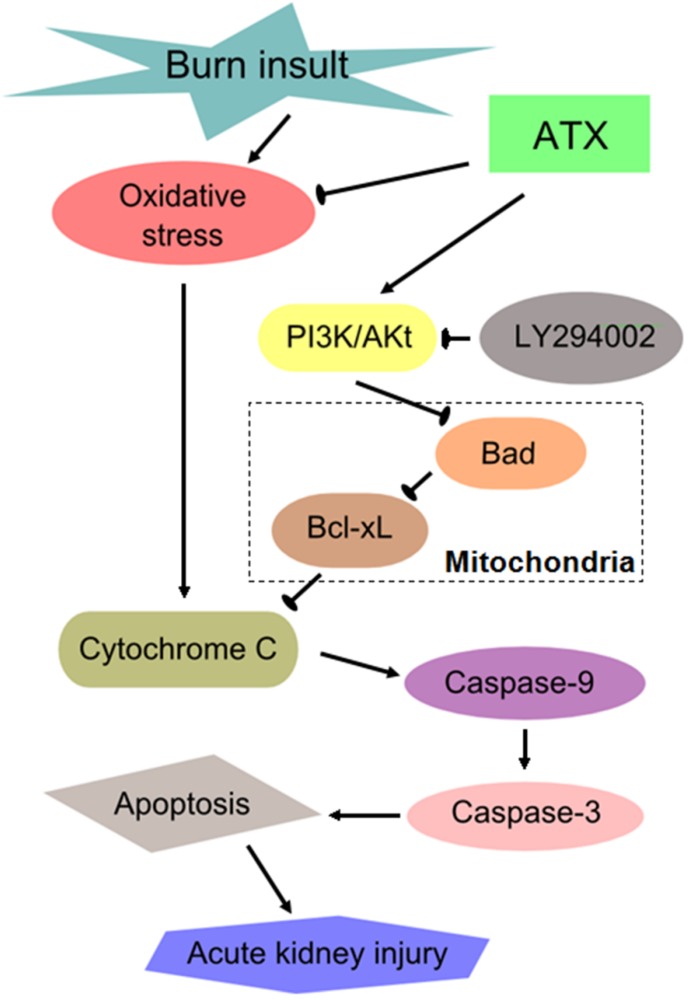
Schematic diagram of the potential mechanisms of burn-induced AKI and the routes by which ATX exerts its effects. ATX = astaxanthin.

Similar to other severe traumatic injuries, burn insults with large body surface areas (usually TBSA ≥20%) causes a series of secondary remote organ damage effects in addition to the direct thermal injury to local skin [37]. Common devastating complications in critical care patients after severe burns include acute lung injury, myocardial injury, AKI, and sepsis, and these complications are associated with higher morbidity and mortality. There has been a global initiative to reduce the enormous and increasing burden and consequences of AKI, which is a syndrome of abrupt loss of kidney function [38]. Clinical doctors are eager to develop effective ways of preventing and intervening in early AKI in severely burned patients given its poor outcome. The mechanism of early AKI post burn is multifactorial, and previous studies mostly focused on oxidative stress injury, renal tubular apoptosis and systemic or local inflammation [2,3]. Previous results (from our group and others) have shown that ROS-induced oxidative stress injury and apoptosis play important roles in the development of burn-induced early AKI and that 24 h post burn is a significant time-window for observing the changes in renal function and the levels of oxidative stress and apoptosis [18]. In the present study, we investigated the protective effect of ATX on early AKI in severely burned rats, and discovered its dose-dependent benefit on possible AKI prevention, which may be mediated by ameliorating the oxidative and mitochondria-related apoptosis (Figure 8).

ATX can be extracted from ocean crustaceans, such as shrimp, crawfish, crabs and lobster, and has been approved by U.S. Food and Drug Administration as a feed additive and dietary supplement since 1987 and 1999, respectively [39]. Its molecular structure makes it easy to move through physiologic barriers, even the blood-brain barrier, and cell membranes [40]. Many benefits in several diseases have also been discovered based on the results of *in vivo* and *in vitro* studies [23,28,30,33,34]. Considering the difficulty of oral intake in severely burned patients due to pharyngolaryngeal injury and impaired conscious, intravenous injection was selected as an effective and convenient route of medication administration. The ATX doses and preparation method were selected based on the pharmacokinetics findings of Choi HD and colleagues [25]. The results of renal histological and functional evaluation in the burn models indicated that ATX attenuates severe burn-induced tubular injuries and reduces the elevation in the Cr and NGAL levels, which reflect the alteration of renal function and represent sensitive indicators or biomarkers of AKI [8,18,41,42,43]. It was also observed that the effect of ATX enhanced gradually with increasing doses. Then, we explored the potential mechanism of ATX action.

ATX is a natural and powerful antioxidant, and exhibits antioxidant activity that is ten times higher than other biomolecules, such as canthaxantin, b-carotene and zeaxanthin [30]. Regarding its antioxidant properties involved in the quenching of ROS and free radicals, several studies has investigated its therapeutic value in traumatic stress, diabetes, cardiovascular diseases and tumorigenesis [12,23,30,33,34]. Previous evidence has suggested that ROS-induced oxidative stress plays a vital role in the pathogenesis of early AKI and other burn-induced organ injuries [10,44,45,46,47]. Moreover, the kidney, which contains a large number of polyunsaturated fatty acids, is an organ that is vulnerable to ROS-mediated oxidative stress [48,49]. To assess the effect of ATX on oxidative stress, we selected OPR and MDA as direct indices to indicate the overall redox status [50,51]. In addition, SOD and CAT, which are common enzymes of the endogenous antioxidant system, are generously consumed under conditions of increased oxidative stress [43,44,52]. Therefore, their activities represent the extent of oxidative stress. In our study, we found that ATX administration effectively decreases the elevated ORP value and MDA levels in the kidneys of rats after severe burn. In addition, ATX also significantly up-regulated the activities of SOD and CAT. ATX showed dose-dependent effects that peaked at the 20 mg/kg dose. These data indicated that ATX protects against early AKI post burn by relieving ROS-induced oxidative stress as well as restoring the suppressed activities of endogenous antioxidant enzymes.

The kidney usually undergoes tubular apoptosis as a response to various insults, such as burns, ischaemia, radiation, trauma, or toxic injury [31,53,54,55,56]. Previous research also demonstrated key roles of apoptosis in renal functional alterations and prognosis in patients after burn injury [54]. Thus, the regulation of renal tubular apoptosis might be a critical target for preventing or improving early AKI post burn. The results of TUNEL staining exhibited obvious augmentation of apoptotic tubular cells after burn, which was consistent with our previous study. With increased doses of ATX, the number of apoptotic renal cells decreased substantially, which validated our hypothesis regarding the effect of ATX on ameliorating tubular apoptosis. We also observed an interesting result that LY294002, a classic inhibitor of PI3K, almost completely reversed the high-dose ATX-induced reduction in apoptosis in the renal tissue post burn, which proposed the possible involvement of the PI3K/Akt pathway and their downstream mitochondrial-apoptotic signalling cascades in the mechanism of ATX.

The classic PI3K/Akt signalling pathway plays an important role in antagonizing renal tubular apoptosis caused by burn insults [18]. Activated Akt (p-Akt) further phosphorylates downstream signalling molecules, such as Bad and Bcl-2 associated X protein (Bax), which results in the inhibition of their activities and binding abilities and further reduces the expression of pro-apoptotic proteins [57]. Jiang L *et al.* demonstrated that the over-expression of Bad induces apoptosis via a mitochondrial-dependent pathway in non-small cell lung cancer [19]. Previous researchers also suggested that Bad, a member of the Bcl-2 family, associates with Bcl-xL in the mitochondria to form a pro-apoptotic complex to further influence the release and activation of downstream pro-apoptotic proteins, such as cytochrome c and caspases [58,59]. Increased p-Akt causes Bad to dissociate from Bcl-xL through a serine-136 phosphorylation and reduces caspase-dependent apoptosis [58,59]. Cytochrome c is considered to be a non-invasive biomarker of AKI and is a new target of mitochondrial-targeted therapeutics because its release indicates mitochondrial damage and apoptosis in the early stages of AKI [60]. Cleaved caspase-3/9 represents the activated status of caspase-3/9, as a result of increased cytochrome c, and is involved in a mitochondrial-dependent pathway that eventually leads to apoptosis [58,59]. To elucidate the regulatory details of ATX application, we investigated the entire Akt/Bad/Caspase signalling cascade and discovered that ATX increases Akt and Bad phosphorylation, which resulted in further decreased expression of cytochrome c and caspase-3/9. Combined with the TUNEL staining results, we presumed that ATX attenuated burn-induced early AKI by regulating mitochondrial-related apoptosis. LY294002 pretreatment largely reversed the protective effect of ATX on renal tubular apoptosis after burns in rats, as well as the expression of signals in the mitochondrial-dependent pathway, which supported our deduction about the implication of the Akt/Bad/Caspase signalling cascade in the nephroprotection of ATX in early AKI secondary to severe burns.

Although we obtained valuable data about the benefits and mechanisms of ATX in preventing early AKI after severe burns, our study still has some limitations. First, the selected ATX doses are specific to the rat model, and the suitable doses for humans require clinical studies. Additionally, we chose the venous pathway based on our clinical experience; however, other routes of administration should be investigated as alternatives. Except Bad, other downstream pro-apoptotic proteins may be involved in the PI3K/Akt regulation of apoptosis, such as Bax and glycogen synthase kinase-3 (GSK3), whereas the inflammation signalling pathway may also be included in the mechanism of ATX. Lastly, we did not evaluate the details of ATX on free radicals and the possible effects on normal metabolism based on its extremely powerful antioxidant activity. Our further works will focus on these issues.

## 4. Materials and Methods

### 4.1. Animals

Adult male Sprague-Dawley rats (weighing approximately 220–250 g) were purchased from the Animal Centre of Zhejiang Chinese Medical University (Hangzhou, China) and were housed on a 12-h light/dark cycle in an air-filtered unit with consistent temperature and humidity and free access to food and water. All experiments performed in this study were approved by the Zhejiang University Committee on Animal Care and strictly abided by the National Institutes of Health Guidelines for the Care and Use of Laboratory Animals (The ethical committee approval number is 2014-140).

### 4.2. Severe Burn Model and Experiment Groups

**Figure 9:**
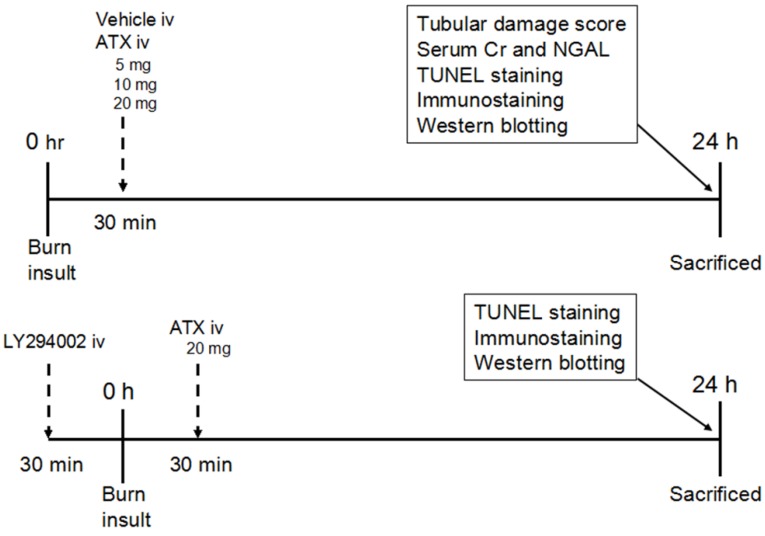
Schematic illustration of the experimental design and animal group classification. ATX = astaxanthin, Cr = creatinine, iv = intravenous injection, NGAL = neutrophil gelatinase-associated lipocalin, TUNEL = terminal deoxynucleotidyl transferase-mediated dUTP nick-end labelling.

After intraperitoneally injecting (ip) sodium pentobarbital (50 mg/kg), the severe burn rat model was produced by exposing the rats to a 15 s immersion into 100 °C hot water to generate a full thickness dermal burn with 40% TBSA [61]. The sham group was treated with an exposure to 25 °C water after anaesthesia [61]. Liquid resuscitation with lactated Ringer solution (LRS) at 4 mL/kg/TBSA was administered via an IP injection immediately and 6 h after the operation. The breath and heart rate of burn rats were carefully monitored to ensure that all rats were under anesthetic and painless before recovering from anesthesia. In addition, rats were housed in individual cages and administered 0.25 mg/kg of buprenorphine by subcutaneous injection immediately and every 12 h post burn for analgesia. The pain and distress scale (Table 1), reported previously, were conducted immediately and every 6 h after recovering from anaesthesia to evaluate the pain condition of rat models and instruct pain-reliving therapy [62]. If the score was higher than 2 in any category, and/or if the total score was higher than 3, we consulted the veterinarian for additional analgesia, warming, or other appropriate measures (e.g., immediate euthanasia). Furthermore, rats were provided easy access to food and water after analepsis when the score in appetite was higher than 1. The design of this study is shown in Figure 9. Eighty-four animals were randomly divided into seven groups: sham group (*n* = 12), burn group (*n* = 12), burn plus vehicle group (*n* = 12), burn plus 5 mg/kg ATX group (*n* = 12), burn plus 10 mg/kg ATX group (*n* = 12), burn plus 20 mg/kg ATX group (*n* = 12), and burn plus 20 mg/kg ATX plus LY294002 group (*n* = 12). In the sham and burn groups, no special drugs were administered after the hot water exposure. The animals in the various burn plus ATX groups received ATX (Sigma-Aldrich, St. Louis, MO, USA) (dissolved in polyethylene glycol 400-*N*,*N*-dimethylacetamide (PEG400) purchased from Sigma-Aldrich, St. Louis, MO, USA (50:50, v/v)) at doses of 5, 10, and 20 mg/kg via tail intravenous injection (iv). Equal volumes of the vehicle (PEG400 (50:50, v/v) without any drugs) were administered to the rats in the burn plus vehicle group. In addition to ATX injection, the burn plus 20 mg/kg ATX plus LY294002 group were also administered LY294002 (Sigma-Aldrich, St. Louis, MO, USA) (dissolved in PEG400 (50:50, v/v)) at a dose of 0.3 mg/kg. The doses and administration method of ATX and LY294002 were selected according to previous studies [25,63]. All animals were sacrificed at 24 h by overdose of sodium pentobarbital after the procedure based on our prior study. Both kidneys were dissected after cardiac perfusion with phosphate-buffered saline (PBS) (pH = 7.2) and were maintained in 10% formalin at 4 °C or in a −80 °C freezer for subsequent experiments.

**Table 1:** The pain and distress scale used in the present study.

Assessment Score	0	1	2	3
**Attitude and posture**	Alert and not hunched	Not alert or hunched	Not alert and hunched	Not responsive to stimuli
**Gait and movement**	Active	Somewhat inactive	Completely inactive	Lying on side
**Burn wound site**	Clean and not swollen	Exudates or swelling	Exudates and swelling	Increased wound depth in examination
**Appetite**	Eating and drinking	Reduced eating or drinking	Not eating or drinking	Not eating or drinking for >3 days
**Elimination**	Normal	Softer than normal	Diarrhea	Diarrhea >3 days

### 4.3. Histological and Renal Function Evaluation

The fixed kidney samples were cut into 7-μm-thick sections for HE staining, and the tissue slices were observed under a microscope (DM2500, Leica, Solms, Germany). The tubular damage score was determined based on the percentage of renal cortical tubules that expressed epithelial necrosis and was ranked as 0: normal, 1: less than 10%, 2: 11 to 25%, 3: 26 to 75%, and 4: greater than 75%. Eight high magnification files were randomly selected for observation from four slices per group in a blind manner. Blood samples were collected to measure the serum Cr levels via a clinical chemistry analyser system and kits (7180, Hitachi High-tech, Tokyo, Japan). The serum NGAL levels were detected in the various groups using a Rat NGAL ELISA kit (Boster, Wuhan, China) according to the manufacturer’s instructions.

### 4.4. Assessment of Oxidative Stress

The ORP value was determined using the HI3131B electrode (Hanna Co., Ltd., Rome, Italy), according to the provided instruction; 0.5% renal tissue homogenate was injected into the device under airtight conditions at 25.2 °C for detection. The renal tissue homogenate was reacted with a thiobarbituric acid reactive species (TBARS) assay kit (KeyGEN, Nanjing, China) to obtain the MDA levels. Tissue SOD and CAT activities were measured using commercial assay kits from KeyGEN Biotech (Nanjing, China) according to the manufacturer’s protocols. The absorbance values were measured using a microplate reader (Model 680 Microplate Reader, BIO-RAD, Hercules, CA, USA).

### 4.5. TUNEL Staining for Apoptosis

TUNEL staining was performed via a commercial cell death detection kit, purchased from Roche Diagnostics (Indianapolis, IN, USA). The slices were observed and photographed under a microscope (DM5500B, Leica, Solms, Germany). The apoptotic index was calculated as the percentage of apoptotic cells *vs.* the total number of cells counted in a blinded manner.

### 4.6. Immunohistochemistry (IHC) Staining

Paraffin-embedded tissues were cut into 5-μm-thick slices for IHC examination. The sections were incubated with anti-p-Akt (1:200) and anti-p-Bad (1:200) antibodies (both from Cell Signaling Technology, Boston, MA, USA) overnight at 4 °C. Then, they were incubated with goat anti-rabbit secondary antibody (Boster, Wuhan, China), and visualized with a 3,3-diaminobenzidine (DAB) kit (Boster, Wuhan, China). Finally, the mounted sections were observed and photographed under a microscope at 200× magnification (DM2500, Leica, Solms, Germany).

### 4.7. Western Blotting Analysis

The right kidneys of the rat models were maintained for western blotting analysis. Briefly, the frozen renal tissue samples were cut into pieces and lysed with RIPA lysis buffer (AR0105, Boster, Wuhan, China) for one hour on ice, and the lysates were centrifuged at 14,000 *g* for 10 min. Mixed with loading buffer, the protein samples were subjected to SDS-PAGE and transferred onto nitrocellulose membranes by electrophoresis, while aliquots of the coped samples were used to determine the protein concentrations of each sample with a BCA kit (KGPBCA, KeyGEN Biotech, Nanjing, China).The transferred membranes were subsequently blocked and incubated overnight at 4 °C with the following primary antibodies: anti-Akt (1:500), anti-Bad (1:1000), anti-Bcl-xL (1:800), anti-Cytochrome C (1:1000) (all from Santa Cruz, CA, USA), anti-p-Akt (1:500), anti-p-Bad (1:1000), anti-Cleaved Caspase-9 and anti-Cleaved Caspase-3 (all from Cell Signaling Technology, Boston, USA). GAPDH (Santa Cruz, CA, USA) was blotted on the same membranes as a control. Blot bands were detected with SuperSignal^®^ West Dura Extended Duration Substrate (Pierce, Rockford, IL, USA) and X-ray Film (Kodak, Rochester, NY, USA) and were then analysed with Bandscan 5.0 software and compared with GAPDH.

### 4.8. Statistical Analysis

The data are presented as the mean ± standard deviation (SD). GraphPad Prism version 5.02 (San Diego, CA, USA) and SPSS 19 (SPSS, Chicago, IL, USA) were used for the statistical analysis. An unpaired *t*-test was used for comparisons between two groups compared. Multiple comparisons were analysed with one-way analysis of variance (ANOVA) followed by a Student-Newman-Keuls test. A value of *p* < 0.05 was accepted as statistically significant.

## 5. Conclusions

In conclusion, the present study first demonstrates the protective effects of ATX against early AKI following severe burns in rats. The therapeutic effects of ATX increase in a dose-dependent manner. The beneficial effects are due to its abilities to relieve oxidative stress and inhibit apoptosis by modulating mitochondrial-apoptotic pathways, such as the Akt/Bad/Caspase signalling cascade. Thus, ATX will hold great potential as a new drug for the treatment of severely burned patients with early AKI.
